# Medical Advertising

**Published:** 1855-08

**Authors:** 


*Medical Advertising.*—Prof. Davis, in the North Western Medical and Surgical Journal, for May, 1855, refuses to publish the advertisement of the New York University, on the ground that by so doing he would become *particeps criminis* in a falsehood. We have not the room to publish his entire article, neither do we wish to do so, as we have a sincere respect for the *recent* additions to the faculty of that school, but we do wish to endorse the action of Prof. Davis, and profess our determination to be governed by the same principles.

He says: "We have recently received several advertisements, with a request that they should be inserted in the Journal. If we were willing to be governed by the principles which seem to govern the newspaper press generally, we should insert them all without the least hesitation. We have ever doubted the correctness of the principle, however, and shall entirely repudiate it, so far as our own journal is concerned.

"We shall publish no advertisements *merely* because they can be paid for. According to our ideas of morality, a publisher who for a stipulated price, inserts an advertisement in his paper or journal, becomes, in part, at least, responsible for the influence it may exert on his readers. It is in vain that the publisher attempts to shield himself under the plea that the advertiser is alone responsible for the influence of his advertisement; that his paper is only the *passive* medium through which it comes before the public. For it requires only a very limited observation to show that a large class of readers indissolubly connect the character of the editor and publisher with everything they find in their columns. And the expression is not uncommon, that there must be some truth in such and such statements or advertisements, or Mr. A. and Mr. G. would not have published them."

There is good sense, honesty, and logic, in that brief paragraph. Neither is Prof. Davis alone in his manly protest against collegiate quackery. The Stethoscope (Richmond, Virginia,) enters the following protest:

"We shall retain the liberty of rejecting advertisements which savor in any degree of the pretensions of the quack or the self-laudations of the mountebank.

"Our present number contains the announcement of a medical school, (viz., University of New York,) with its curriculum of study, which appears to us to be liable to just and severe criticism: 'The session of 1854–55 was attended by a class of three hundred and seven students, on one hundred and six of whom the degree of doctor of medicine was conferred.'

"To our old-fashioned notions of propriety, this is a *strange* preface for the announcement of a term of instruction in a scientific institution.

"We came to the conclusion that the University of New York is the place in which a diploma may be very easily obtained; and this was no doubt the impression designed to be created.

"We trust that the deans of medical faculties, who send us advertisements, will in future be careful to omit all such appeals.

"We are determined that whatever of influence our journal may possess, shall be thrown (advertising sheets and all) in favor of such institutions as are zealously laboring for more comprehensive courses of instruction, and consequently for an increase of the requirements for the doctorate."

We rejoice in these indications of honor and manly spirit in the periodical press. The profession cannot go far wrong with such guardians. We have noticed a marked change in the tone of the journals during the time that we have been one of the corps editorial. So strong was the conservative tendency but a little while ago, so intense the disgust at the "cut and slash" abusive style of medical writing, that editors generally went to the other extreme, and carefully avoided all censorious comment. But there can be no fears of the prevalence of a scandalous press while such men as Davis and others control it—what we wish to see—what in very many of our exchanges we do see—is a bold, out-spoken spirit, fearless to censure the wrong, but free from personal malice.

